# Centenary of the Medical Society of the State of New York

**Published:** 1906-01

**Authors:** 


**A Monthly Review of Medicine and Surgery.**

*EDITOR:*

WILLIAM WARREN POTTER, M. D.

All communications, whether of a literary or business nature, books for review and exchanges, should be addressed to the editor 284 FRANKLIN ST., BUFFALO, N. Y.

VOL. LXI. JANUARY, 1906. NO. 6

### Centenary of the Medical Society of the State of New York.

IT has fallen to but few societies in America,— to still fewer medical societies,— to attain their hundredth anniversary. It is quite in keeping with the proprieties of such an event that unusual ceremony should be observed, as is proposed by our veteran medical organisation. We use the word "our" in its most comprehensive sense, for it is not alone a society of doctors, by doctors, and for doctors, but it is a society in which eight milllion people take interest, a goodly number of whom will cast their thoughts toward Albany during the days of the Centennial meeting,—January 30, 31 and February 1,—and wish the society well which has so faithfully guarded the public and private health interests for a hundred years.

The occasion is one for reflection; much could be written of achievment, of dissapointment, of vain hopes, of triumphs of joy and of sorrow. But in this busy age, in this "iron age of positivism," it is doubtful if what we should write would be read with patience were we to indulge in sentiments of this kind. Let us rather turn our attention to the immediate present, and give some detaile of the ceremonies which the officers of the society are preparing for the occasion.

The Centennial celebration committee has been at work for nearly a year in preparing some special features for the meeting.

On Tuesday evening, January 30, the president, Dr. Joseph D. Bryant, will delliver an address at the Baptist Church, after which other addresses will be made by prominent speakers. An oration on Medicine by Dr. Samuel B. Ward, and one on Sanitation by Dr. Hermann M. Biggs, will be delivered on Tuesday afternoon; and on Wednesday afternoon Dr. Roswell Park will deliver an oration on Surgery, at the same place. Addresses also are expected from Dr. L. S. McMurtry, president of the American Medical Association, Dr. William H. Welch, of Baltimore, Dr. Charles A. L. Reed, of Cincinnati, and others.

The scientific sessions will be held at Odd Fellows Hall, on Lodge Street, which will occupy the mornings, and for which an ample program is preparing. A Centennial banquet will be served at Odd Fellows Hall Wednesday evening at 7 o'clock, which will be made worthy of the historic event. Distinguished after-dinner speakers will be present and contribute to the festivities. The price of each plate is $5.00, and those who wish to be present should remit this sum to the chairman of the committee on arrangements, Dr. W. J. Nellis, 210 State Street, in order to obtain proper assignments of seats, as the number of banqueters will be very large.

The centennial celebration committee has in preparation a centennial history of the Medical Society of the State of New York, including public addresses delivered at the Centennial Meeting by Hon. Grover Cleveland, Hon. St. Clair McKelway, Dr. Joseph D. Bryant, Dr. S. B. Ward., Dr. H M. Biggs, Dr. Roswell Park, Dr. W. H. Welch, Dr. Lewis S. McMurtry, and others. It is issued by subscription only, at the price of $1.75 a copy. Orders should be placed at once, as the edition is limited, with Dr. George Ryerson Fowler, Secretary, 301 De Kalb Avenue, Brooklyn, N. Y., inclosing the money.

It will be seen from the foregoing that this meeting will be one of the most interesting the society has ever held, and no physician who attends will ever regret being present at the first centenary of this great society. The interest of the occasion will be increased and accentuated by the fact that the amalgamation will be officially accomplished at that time, and medical unity in the empire state will be restored. The eyes of all America will be upon us; let us unite to make ourselves worthy of this distinction.

---

THE presidents of the medical organisations of Erie County named below held a smoker on Friday, Jan. 19, 8:30 P. M at the University Club. The project to create a new College of Arts and Sciences in this city was discussed freely and informally. Short addresses were made by members of the profession, representing the several different interests. The meeting was called in the hope of obtaining the opinions and exciting the interest of the physicians of the vicinity, and it seemed to have served its purpose in admirable fashion. A lunch was served. Medical Society of the County of Erie, A. H. Briggs; Erie County Medical Association, A. G. Bennett; Homeopathic Medical Society, E. P. Hussey; Physicians League, Jane W. Carroll;

Academy of Medicine, H. U. Williams; Medical Club, P. W. Van Peyma; Alumni Ass'n., University of Buffalo, F. Thoma; Medical Union, S. S. Greene; Roswell Park Medical Club, T. H. McKee; Esculapian Club, Charles J. Reynolds; Medical Journal Club, H. R. Gaylord; Clinical Club, A. D. Carpenter ;Physicians Club, James S. Porter; Buffalo Ophthalmological Club, Frederick Lewis. About $20,000 was subscribed as folows:

Floyd S. Crego, $1000; Roswell Park, $1000; Lucien Howe, $1000; Charles Cary, $1000; Charles G. Stockton, $1000; M. D. Mann, $1000; Henry R. Hopkins, $500; H. U. Williams, $500; Dewitt H. Sherman, $500; Charles Sumner Jones, $500; Allen A. Jones, $500; Lee H. Smith, $500 ;Ernest Wende, $500; A. W. Hurd, $500; Eugene Smith, $500; DeWitt C. Greene, $300; C. C. Frederick, $250; J. W. Putnam, $250; A. J. Colton, $200; F. C. Busch, $200; DeWitt G. Wilcox, $200; F. Park Lewis, $200; A. L. Benedict, $150; Joseph Fowler, $100; W. C. Phelps, $100; Willis G. Gregory, $100; E. J. Kiepe, $100; B. J. Bixby, $100; A. G. Bennett, $100; R. R. Ross, $100; I. P. Lyon, $100; W. P. Twitty, $100; Irving W. Potter, $100; Charles A. Bentz, $100; A. H. Briggs, $100; N. G. Russell, $100; C. R. Borzilleri, $100; W. W. Plummer, $100; E. H. Long, $100; Max Breuer, $100; W. G. Taylor, $100; C. A. Brownell, $100; T. H. McKee, $100; A. T. Lytle, $100; T. M. Leonard, $100; T. P. Trevett, $100; F. W. Filsinger, $100; H. R. Trick, $100 ;F. E. Fronczak, $100; Fridolin Thoma, $100; J. A. Gibson, $100; Grover Wende, $100; Edward Clark, $100; A. W. Bayliss, $100; L. E. Curtice, $50; J. R. Gray, $50; F. W. Barrows, $50; R. Meisenbach, $50; A. W. Hengerer, $50; A. E. Hubbard, $50; Jane W. Carroll, $50; Helen Kuhlman, $50; M. M. Huntley, $50; M. Olson-Woods, $50; Ida C. Bender, $50; E. W. Jones, $50; F. J. Parmenter, $50; J. B. Frisbee, $50; W. T. Getman, $50; William Gertner, $50; E. L. Frost, $50; Electa C. Whipple, $50; Thew Wright, $50; M. Elizabeth Schugens, $25; Lillian C. Randall, $25; Bernard Cohen, $25; H. M. Weed, $25.

A committee was appointed to visit those physicians who were not present, which will result, it is expected, in doubling this amount.

---

The New York Medical Journal, which lately incorporated with it The Philadelphia Medical Journal, and still more recently The Medical News, issued a handsome edition January 6, 1906, in commeration of the last named event,—namely, the amalgamation of the Medical News. The number referred to contained 64 pages of reading matter proper, which began with a contribution by Dr. John B. Murphy, of Chicago, and continued with a number of other authors of national reputation. Prize essays, therapeutic notes, editorial material, society proceedings, pith of medical literature, news notes, book notices, and other material followed, the whole making one of the most interesting weekly medical magazines that has been issued of late by any publisher. The advertising columns, too, were replete with fine quality from among the best known houses in the country and the 82 pages in this department could be examined with interest, not to say profit, by every reader of this great journal. The cover in blue tint served to accentuate this extraordinary edition.

